# Cyclin-dependent kinases and CDK inhibitors in virus-associated cancers

**DOI:** 10.1186/s13027-020-00295-7

**Published:** 2020-05-01

**Authors:** Shaian Tavakolian, Hossein Goudarzi, Ebrahim Faghihloo

**Affiliations:** Department of Microbiology, School of Medicine, Shahid Beheshti University of Medical Sciences, Tehran, Iran

**Keywords:** CDK, CIKs, Cancer, Virus

## Abstract

The role of several risk factors, such as pollution, consumption of alcohol, age, sex and obesity in cancer progression is undeniable. Human malignancies are mainly characterized by deregulation of cyclin-dependent kinases (CDK) and cyclin inhibitor kinases (CIK) activities. Viruses express some onco-proteins which could interfere with CDK and CIKs function, and induce some signals to replicate their genome into host’s cells. By reviewing some studies about the function of CDK and CIKs in cells infected with oncoviruses, such as HPV, HTLV, HERV, EBV, KSHV, HBV and HCV, we reviewed the mechanisms of different onco-proteins which could deregulate the cell cycle proteins.

## Introduction

Cell division is controlled by various elements [1–10], especially serine/ threonine protein kinase complexes, called cyclin-dependent kinases (CDKs), and cyclins, whose expression is prominently regulated by the binding to CDK inhibitors [11, 12]. In all eukaryotic species, these genes are classified into different families. It is well-established that the complexes of cyclin and CDK could regulate the distribution of cells in different phases of the cell cycle through modulating the transition of cells toward each phase. By constructing a complex with cyclinE and cyclinA, CDK2 facilitates the progression of S phase. Much of our recent knowledge about the significant role of CDKs and CDK inhibitors is emanated from studying RbyE2F pathway, which resulted in the discovery of the principal substrates of these proteins, such as Rb, p107, p130, E2F-1, and DP-1 [13–15]. It has been investigated that when these mentioned substrates are phosphorylated, CDKS bind tightly to their especial motif (the RXL motif) [16–18]. This finding highlights the key role of the phosphorylation in the entrance of the cells to the S phase of the cell cycle [19].

CDK genes are classified in mammalian cells into different classes of CDKs, especially some important regulatory ones (The regulatory CDKs play important roles in mediating cell cycle). Each of these CDKs could interact with a specific cyclin and thereby regulating the expression of different genes [20, 21]. Classical cell cycle CDKs, Cdk4, Cdk6, Cdk2 and Cdk1 regulate the transitions through the different phases of the cell-division cycle, and activating of these genes are at least partially mediated by the control of multiple transcription factors (TFs) or regulatory elements such as the retinoblastoma protein (Rb). The other group of regulatory CDKs includes CDKs 10, 11, 12, 14, 40 16, 19, 5, 7, 8, and 9. Cdk10 and Cdk11 control transcription by phosphorylating TFs, hormone receptors and associated regulators (HRs), or splicing factors (SPFs) while Cdk7, Cdk9 and Cdk12 directly phosphorylate the C42 terminal domain (CTD) of RNA polymerase II (RNAPII), thus modulating the different phases of generation of transcripts. The Mediator complex is specifically regulated by Cdk8 or the highly related Cdk19. Cdk7 functions as a CDK-activating kinase (CAK) by directly phosphorylating several of the CDKs mentioned above. Cdk5 displays many functions in the cell, but it is better known for its function in the control of neuron-specific proteins such as Tau. The members of the Cdk14 subfamily, such as Cdk14 itself or Cdk16, are activated at the membrane by cyclin Y and also participate in many different pathways, such as Wnt-dependent signaling or signal transduction in the primary cilium. CKI family, comprised from two main genes, particularly targets CDKs through its phosphorylation and halts the transition of cells from different phase of the cell cycle, leading to cell cycle arrest. The first gene family associated with CKIs is INK4 gene family (p16INK4a, p15INK4b, p18INK4c, and p19INK4d), which can interact with CDK4 and CDK6, and prevent their activity. The second family of genes is composed of p21Cip1/Waf1/Sdi1 [22, 23], p27Kip1 [24, 25], and p57Kip2 [26] that can interfere with cyclin D-, E-, A-, and B53 CDK complexes [27]. Sharing a similar N-terminal domain, all these molecules could bind to the cyclins and CDKs; however, given to their different structure, each CKI participates in a distinct cell function [21]. Mounting body of evidence has shown that in cancer cells the alteration of the expression level of CDKs and CDK inhibitors may provide a platform for malignant cells giving them the opportunity to proliferate vigorously. The association between CDKs/CDK inhibitors with viral onco-protiens has been investigated in numerous studies. For instance, a recent report suggested that avian reovirus p17 protein is a virus-encoded CDK inhibitor (V-CDKI) and downregulates CDK7/cyclin H complex [28]. In the present study, we did an intensive literature review to shed light on the underlying mechanisms through which viral onco-protiens regulate the expression of CDKs and CDk inhibitors, leading to tumor progression.

## Human papillomaviruses

**Fig. 1:**
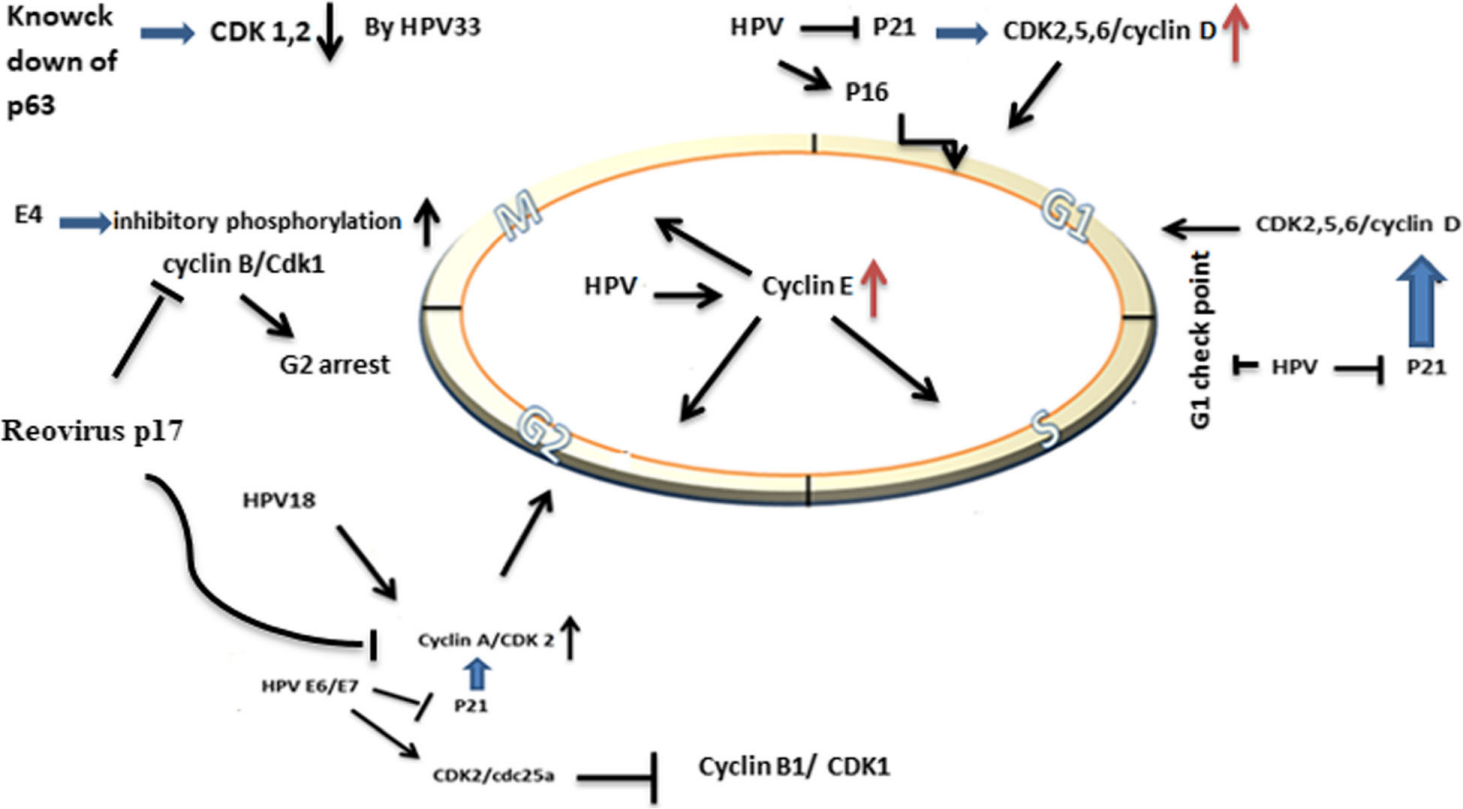
The schematic diagram representing the effects of HPV on CDK and CIKs

Human papillomaviruses, comprising over 100 genotypes, are non-enveloped and double-stranded DNA viruses [29]. More than 30 years ago, it has been assumed that HPV may participate in different types of cancers, including cervical, anus, oropharyngeal cancer. The results of studies indicated that there is a direct relationship between high-risk strains of HPV (HPV16, 18, 31, 33, 45, 52, and 58) and the incidence of human cancers [30]. Once HPV infected skin and mucosal surfaces, this virus not only initiates replication in the undifferentiated, proliferating cells of the basal layer but also through stimulating the host cell to divide, HPV increases its genome widely. Although the underlying mechanisms responsible for HPV replication are not entirely clear, it is suggested that some oncoproteins, such as E6 and E7 are involved in this process [31]. E6 and E7 can deregulate proteins, which control mitosis. After binding to p53, E6 destroys this tumor suppressor protein by exploiting the ubiquitin pathway. It has also been indicated that E7 could separate Rb from E2F and subsequently lead to the cell cycle progression [32]. Moreover, the results of some other studies suggested that HPV onco-proteins could interact with cell cycle regulatory proteins, such as CDKs, and CDK inhibitors. There are some evidence showing that E7 can prevent the activity of tumor suppressor, called p21^Cip1^ in cervical cancer [33]. E7 either expressed in high-risk strains of HPV (HPV16 and HPV31) or in the low-risk strain of the virus (HPV6b) could increase the activity of CDK2. The analysis of E7 protein sequences in these strains revealed that amino acids 9 to 38 participate in the induction of CDK2 activation. It has been reported that the 640 ng Glutathione-S-transferase (GST)–E7 can directly activate CDK2/cyclin A histone H1 kinase. Although GST-16E7 could stimulate CDK5/p25 complex, this protein has no inductive potential on CDC2/cyclin B activity [34]. By binding to the carboxy-terminal end of p21, HPV-16E7 blocks the activity of p21 on proliferating cell nuclear antigen (PCNA)-dependent DNA replication [35]. In addition, HPV-18 E1^E4 can promote both cyclin E and cyclin A/CDK 2 through binding to RXL motif [36]. The activation of CDKs may be up-regulated by HPV-16 E7. HPV-16E7 increases the expression level of cyclin E both transcriptionally and post transcriptionally, stimulating the entrance of cells into S and G2/M phase of the cell cycle [37]. The expression of CKI depends on the context of the cells. Although the expression of CKI is up-regulated in hyperplasia and koilocytes, its expression is down-regulated in cervical carcinoma. The expression of p16 also is increased in neoplastic cells [38]. HPV E6 could regulate the expression of p21 through down-regulation of p53 [39]. By changing the expression of cyclin/CDK, HPVE6 impairs G2 check point, which in turn lead to chromosomal instability [40]. The expression level of both p21WAF1/CIP1 and p27KIP1 can be negatively regulated in cervical lesions [41]. In a study conducted by Cho et al., it has been indicated that while the cyclin E was over-expressed in cervical lesions, the expression of cyclin D was decreased as result of p2lWAFI/CIPI and p27KIP1 down-regulation [42]. The association between HPV E6 and E7 proteins and cell cycle regulatory proteins has been well-established in another study conducted by Kim et al. They reported that treatment of TC-1 cells, expressing both HPV E6 and E7 proteins, with bortezomib and celecoxib could trigger apoptotic cell death through MAPK-mediated suppression of cyclin D1 and CDK2 [43]. The results of precise analysis also showed that knocking down p63 expression in differentiating HPV31 positive keratinocytes decreased the expression of cyclins A, B, and E, as well as Cdc25c, Cdk1 and Cdk2 [44]. Additionally, the high levels of cytoplasmic cyclin B1 and Cdk1 in HPV18 positive keratinocytes may induce G2/M cell cycle arrest [45]. It has also been demonstrated that E7 protein can increase the expression level of CDK2 by interacting with cyclin A, cyclin E, and cdc25a phosphatase [46–48] (Fig. 1) (Table 1).

**Table 1:** The different effects of oncovirus on CDK and CIKs

Viral pathogen	Viral Factor	HDAC/Mechanism/Effect	References
**HPV**	E7	p21Cip1 suppression	Shin MK et al.,2009 [33]
**HPV**	E7	CDK2/cyclin A activation	He W et al.,2003 [34]
**HPV16**	E7	block the effect of p21on (PCNA)-	Funk J et al.,1997 [35]
**HPV-18 E1^E4**	E1^E4	both cyclin E and cyclin A/CDK 2 activation	Ding Q et al.,2013 [36]
**HPV-16**	E7	Cyclin E activation- decrease in CIKs except, p21 and p16	Martin L et al.,1998 [37]
**HPV**	E6	Decrease in p21 and up-regulation of cyclin E/CDK2	Syrjänen SM et al.,1999 [39]
**HPV31**		cyclins A, B, and E, Cdc25c, Cdk1 and Cdk2 activation	Mighty, K.K et al.,2011 [44]
**HPV18**		Increase the levels of cytoplasmic cyclin B1 and inactivation of cyclin-dependent kinase1(Cdk1)	Wang, H.K et al.,2009 [45]
**High**	E7	Promotion of Cyclin A/E/ CDK2	Nguyen, C.L et al.,2008 [46], Katich, S.C et al.,2001 [48]
**HTLV**		Decrease in p21^WAF1/CIP1^	Moles R et al.,2015 [49]
**HTLV**		Down-regulation of p27KIP1	Cereseto A et al.,1999 [50]
**HTLV-I**	Tax	Cyclin A limitation	Kibler KV et al.,2001 [51]
**HTLV**	P30	Binding with cyclin A and cdk2, and preventing enter in S phase	Baydoun HH et al.,2010 [52]
**HTLV**		Increase in cyclin D/ CDK2 and inhibition of INK4	Grassmann R et al.,2005 [53]
**HTLV**		cyclin D2/ CDK4,6	Santiago F et al.,1999 [54]
**HTLV**		p18ink4c inactivation	Suzuki T et al.,1999 [55]
**HTLV**		CDK4	Haller K et al.,2002 [56]
**EBV**	EBNA3C	cyclinA/CDK2 is activation	Knight JS et al.,2004 [57]
**EBV**	EBNA3C	Suppression of p16/INK4a	Parker GA et al.,1996 [58, 59]
**EBV**	EBNA3C	Targeting of SCF/skp2 E3	Kumar P et al.,2009 [60], Iwahori S et al.,2009 [61]
**EBV**	EBNA3C	Repressing the repress p21WAF1/CIP1, p14ARF and p16INK4a	Tursiella ML et al.,2014 [62]
**EBV**	BZLF1	Increase in p21WAF1/CIP1	Sato Y et al.,2010 [63]
**EBV**		Up-regulation of cdc-2, cyclin E, D23, and cyclin D2	Hollyoake M et al., 1995 [64]
**EBV**	LMPA 2A and MYC	Suppress the p27	Kamonwan Fish et al.,2014 [65]
**EBV**		Increase in CDKN2A	Vo QN et al.,2002 [66]
**KSHV**	K-cyclin	CDK6	Li, M et al.,1997 [67]
**KSHV**	K-cyclin	CDK9	Chang PC et al.,2007 [68]
**KSHV**	v-cyclin	P27	Moore PS et al.,2001 [69]
**KSHV**		p21	Van Dross R et al.,2005 [70]
**HBV**	HBx	Suppression of P21	Han,J et al.,2000 [71]
**HBV**	HBX	cyclinA/CDK2	Bouchard M et al.,2001 [72]
**HBV**	HBX	Methylation of P16	Jung JK et al.,2007 [73]
**HCV**	HCV core	Inhibition of CDK7	Ohkawa K et al.,2004 [74]
**HCV**	HCV core	Activation of CDK2	Ohkawa K et al.,2004 [74]
**HCV**	HCV core	cyclinA, E, D1, CDK2 and CDK4	Bahnassy AA et al.,2011 [75]
**HCV**	NS5A	P16,P21,P57	Arima N et al.,2001 [76], Wagayama H et al.,2001 [77], Shackel NA et al.,2002 [78]
**Avian Reovirus**	p17	Activation of v-CDKI	Huang W et al., 2017 [79]
**Avian Reovirus**	P17	CDK inhibitor (V-CDKI)	Kozak R et al., 2017 [80]
**Avian Reovirus**	P17	v-CDKI	Chiu HC et al., 2018 [28]
**Avian Reovirus**	P17	suppression of CDK1/cyclin B1, CDK2/cyclin A2, CDK2/cyclin E1, and CDK6/cyclin D1 complexes by direct binding to CDKs, cyclins or complexes	Huang W et al. 2017 [79]
**Avian Reovirus**	P17	Suppression of the CDK7/cyclin H complex by promoting p53 and cyclin H interaction.	Huang W et al. 2017 [79]

## Human T-lymphotropic viruses and human endogenous retroviruses

Human T-lymphotropic viruses (HTLV-1 and HTLV-2) are among those oncoviruses that are supposed to be involved in different types of cancers. These viruses belong to retroviridae family and are spread worldwide [81, 82] with some endemic areas such as North-Eastern Iran [83, 84]. These viruses possess positive-sense RNA genome that is further converted into DNA by a reverse transcriptase enzyme and then integrated into human genome [85]. It is estimated that 20 millions of people in the world are infected with HTLV; however, they do not display any clinical symptoms in their life time [86]. Basically, HTLV is a congenital infection. It could also be transmitted through blood transfusion, sharing syringe, breastfeeding and unprotected sexual intercourse [87]. TAX and human T-cell leukemia virus type 1 bZIP factor (HBZ) are the most important oncoprotins encoded by HTLV [88]. TAX functions as a trans-activator of viral replication, but it is often inactivated in infected cells [89]. In contrast, HBZ which is responsible for viral latency is expressed in the infected cells [90]. The association between Cyclin-dependent kinase/or Cyclin-dependent kinase inhibitor with HTLV is established in several studies. It has been indicated that the expression level of p21^WAF1/CIP1^ is down-regulated in HTLV infected cells [49]. Moreover, in most ALT and HTLV infected cells, the expression of cyclin E/CDK2 is increased as a result of the suppression of p27KIP1 [50]. By regulating cAMP responses, it has been demonstrated that Tax protein could restrict the activity of Cyclin A promoter [51]. Moreover, HTLV P30 halts the entrance of the cells into the S phase of the cell cycle by binding to cyclin A and cdk2 [52]. As a results of the down-regulation of p21 and INK4 in HTLV infected cells, the expression of cyclin E/ CDK2 and cyclin D/CDK2 is elevated [53]. Of note, the increase in the activity of cyclin D2/ CDK4,6 in infected T cell is coupled with the activation of DNA replication and cell proliferation [54]. Another mechanism by which HTLV1 could increase the expression of CDK4 is mediated through transcriptional suppression of p16 INK4a-mediated p15 INK4b expression. Furthermore, HLTV could bind to the E-Box promoter and thereby repress the expression of p18ink4c in infected cell. HTLV also could increase the expression of CDK4, one of the main regulators of cell growth [55], by directly binding to its N-terminal [91]. Once CDK4 is over-expressed, this kinase can phosphorylate Rb and increase its cellular protein expression level [56]. The association between HTLV and the expression level of CDKs has been reported in cell line studies. It was delineated that HTLV-I infected T-cell lines displayed an over-expression in p21^Waf1/Cip1/Sdi1,^ cyclin D2, p18Ink4, highlighting that HTLV could potently increase the expression of CDK inhibitors in malignant cells [92].

**Fig. 2:**
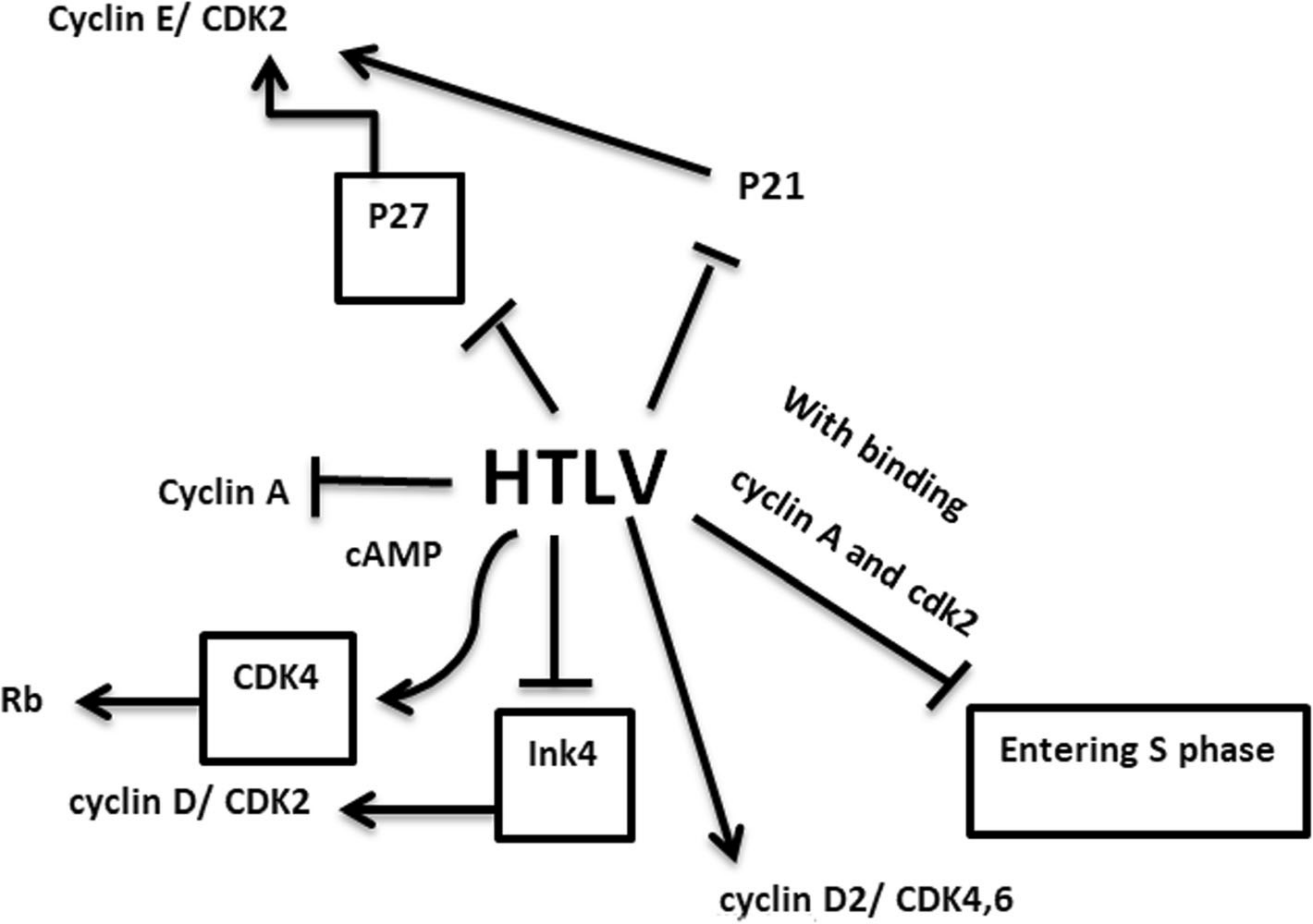
Different mechanisms which show the reciprocal interactions between HTLV and CDK/CIKs in different cancers

Another member of retroviridea family, which has integrated its genome into human germ line about 30–40 million years ago is Human endogenous retroviruses (HERVs). It is estimated that the genome of this virus constructed about 8% of human genome. The over-expression of its association genes has been observed in some types of human cancer [93]. Mounting body of evidence has claimed that HERVs could alter the expression of CDK and CIK. In breast cancer cells, it is demonstrated that HERV-K gene could change the expression level of CDK4 and CDK6 [94]. Moreover, it is reported that HERV-K decreased the expression of p16/CDK4 through BRAF-MEK-ERK signaling pathway in melanoma [95]. (Fig. 2) (Table 1).

## Epstein-Barr virus (EBV)

Epstein-Barr virus (EBV) is one of the etiologic infection agents which is responsible for a wide range of human malignancies, such as Burkitt’s lymphoma, nasopharyngeal carcinoma, post-transplant, AIDS-associated lymphomas, and Hodgkin’s disease [96]. This virus transforms B lymphocytes with EBV nuclear antigen 3C (EBNA3C) into an immortalized cell [97]. It is assumed that EBNA3C could have an impact on the expression of CDK and CKIs, foremost, p16INK4A [58]. Moreover, it has been suggested that by separating p27 from the complex of cyclin A/CDK2, EBNA3C binds to Carboxy-terminus of cyclin A, and increase in the activity of cyclin A/CDK2 complex can be observed [57]. The other mechanism which has been attributed to this virus is mediated through Ras signaling pathway, as EBNA3C could inactivate p16/INK4a and transform embryonic fibroblast. This function is similar to the mechanism exploited by E7 papilloma and E1A adenovirus [59]. During the viral lytic replication, EBNA3C recruits SCF/skp2 E3 ubiquitin ligase and thereby, decreases the amount of p27 in cells which subsequently leads to the activation of cyclin A/CDK2 [60, 61]. As another mechanism, it has been demonstrated that EBNA3C can repress p21^WAF1/CIP1^, p14ARF and p16INK4a, stimulating cell proliferation [62].

**Fig. 3:**
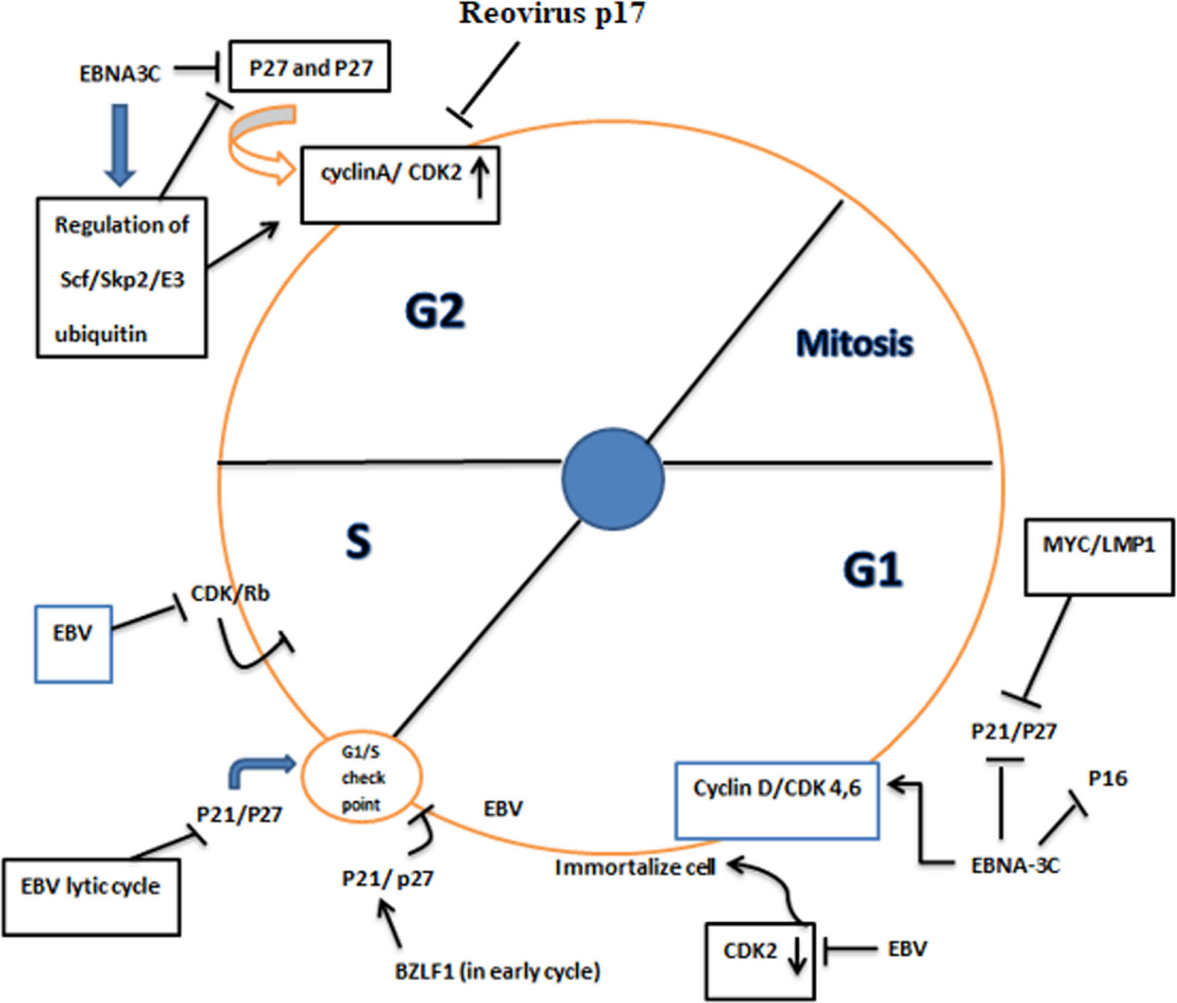
EBV can cause different effects on cyclin and CDK/CLKs

At the early stages of EBV lytic stage, BZLF1, an immediate-early viral gene of the Epstein–Barr virus increases the expression of p21^WAF1/CIP1^ through not only elevating the expression of p53 but also through binding to the promoter of p21^WAF1/CIP1^ [63]. In a study conducted by Hollyoake et al., it has been reported that there is an over-expression in cdc-2, cyclin E, D23, and cyclin D2 in EBV-mediated immortalized cells [64]. EBV can denature and phosphorylate p21^WAF1^ with stabilizing Pim-1 protein in S phase. This virus also can degrade p27KIP1 by modulating the expression of Skp2, one of the subunits of SCF ubiquitin–protein ligase complexes [98]. Moreover, the unique cross talk between EBV latent membrane protein (LMP 2A) and Myc, a family of wide range of onco-proteins, can suppress p27kip1, and facilitate the G1-S transition [65]. Several studies also suggested that there is a tight correlation between the expression of EBV-associated NPC, EBV- LMP-1 and EBER-1 and p16, CDK4, cyclin D1, and Rb. The significant role of aforementioned proteins in different malignancies, including colorectal and nasopharyngeal carcinoma is well-established [99]. Moreover, EBV infection is associated with the increment in the expression of CDKN2A in gastric cancer [66] (Fig. 3) (Table 1).

## Herpes virus type 8 (KSHV)

**Fig. 4:**
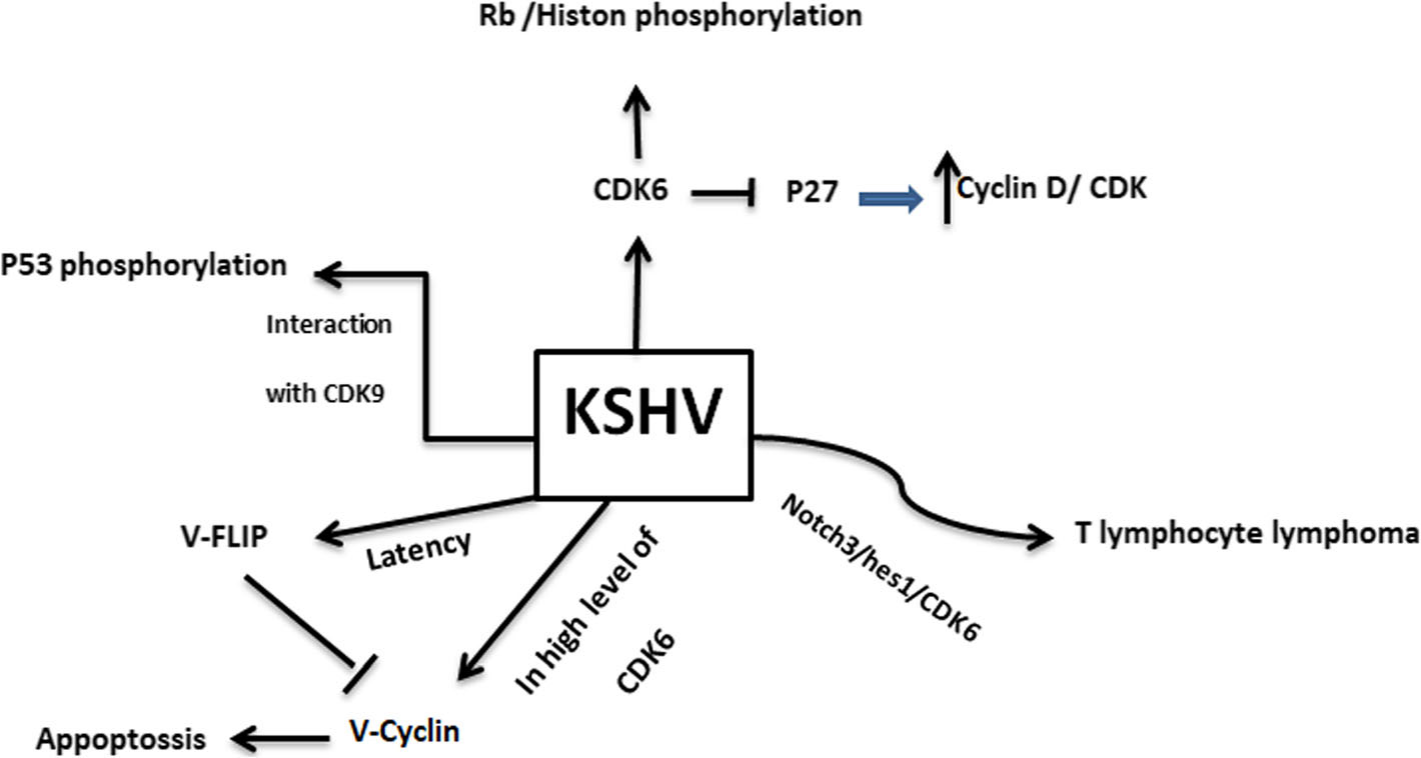
herpes virus type 8 (KSHV) regulates the expression of CDK and CIKs

Herpes virus (KSHV), or human herpes virus 8 (HHV-8), is one of the members of herpes viridea family that accounts for the development of malignancies in patients suffering from AIDS [100]. This virus could also cause non-Hodgkin B-cell lymphoma (PEL), [101] and lymphoproliferative disorder (MCD) [102]. Sharing 30% similarity between K-cyclin, expressed by KSHV, and D-type cyclins leads to the interaction between these two proteins [103]. K-cyclin is able to make a complex with Cdk6, phosphorylate Rb and histone H1 in both G1 and S phases of the cell cycle. This function is similar to the activity of cyclinA/CDK2. Also p27 is one of the main targets of K-cyclin [67]. Moreover, it is suggested that K-cyclin and CDK9 can suppress the activity of p53, through its phosphorylation, leading to tumor progression [68]. Although K-cyclin is considered as an onco-protein, this protein can also lead to the induction of apoptosis in the high level of CDK6 [104]. Integrating with Notch3 and Hes1 signaling pathways, KSHV can interfere with T cell maturity, and initiate lymphoma [105, 106]. HHV-8 can cause primary lymphomas through suppression of p27KIP1 via Ser10 and Thr187 phosphorylation [107]. Dross et al. also confirmed that KSHV could exert an inhibitory effect on the expression of p21 and p27KIP1 [70]. During latency phases, ORF 72 expresses v-cyclin, a homologue of D-type cyclin, which is associated with CDK2, CDK4, CDK9. Indeed, nucleophosmin can be phosphorylated by V-cyclin CDK6, and control the latency phase [108]. Furthermore, it has been reported that v-cyclin-CDK6 is not sensitive to the Cip/Kip and INK4, and can phosphorylate Rb protein and Cdc6 [109]. It is assumed that v-cyclin may inhibit p27 and directly phosphorylate H1 histone [69]. In contrast, V-Flip can prevent the activity of V-cyclin, and induce the autophagy [110] (Fig. 4) (Table 1).

## Human hepatitis B virus (HBV)

Human hepatitis B virus (HBV) is one of the major causes of hepatic disease all around the world. Severe liver disease can also be occurred by co-infection of other types of hepatitis viruses [111–114]. By having a DNA genome, HBV encodes a wide variety of onco-proteins which can deregulate the expression of CDK and CIKs. Hepatitis B virus X protein (HBx) is one of these proteins that affect the pregenome transcription and HBV replication [115]. HBx possesses two important sites, Ser-101 and Met-130, which can activate and repress this protein, respectively. Although the signaling pathway through which HBx regulate the expression of cell cycle regulatory proteins has not been elucidated, some studies have suggested that HBx has a tight association with p21 [71, 116]. It is assumed that Met-130 represses the expression of p21 through suppression of the cellular transcription factor, Sp1 [117]. While several studies claimed that Hbx could repress the expression of p21, other reports suggested that HBx increases the expression of this CDK inhibitor [118]. Src tyrosine kinases-mediated activation of cyclinA and consequently CDK2 may be another mechanism through which HBx may regulate cell proliferation and transition of cells from G1 to S phase of the cell cycle [72].

**Fig. 5:**
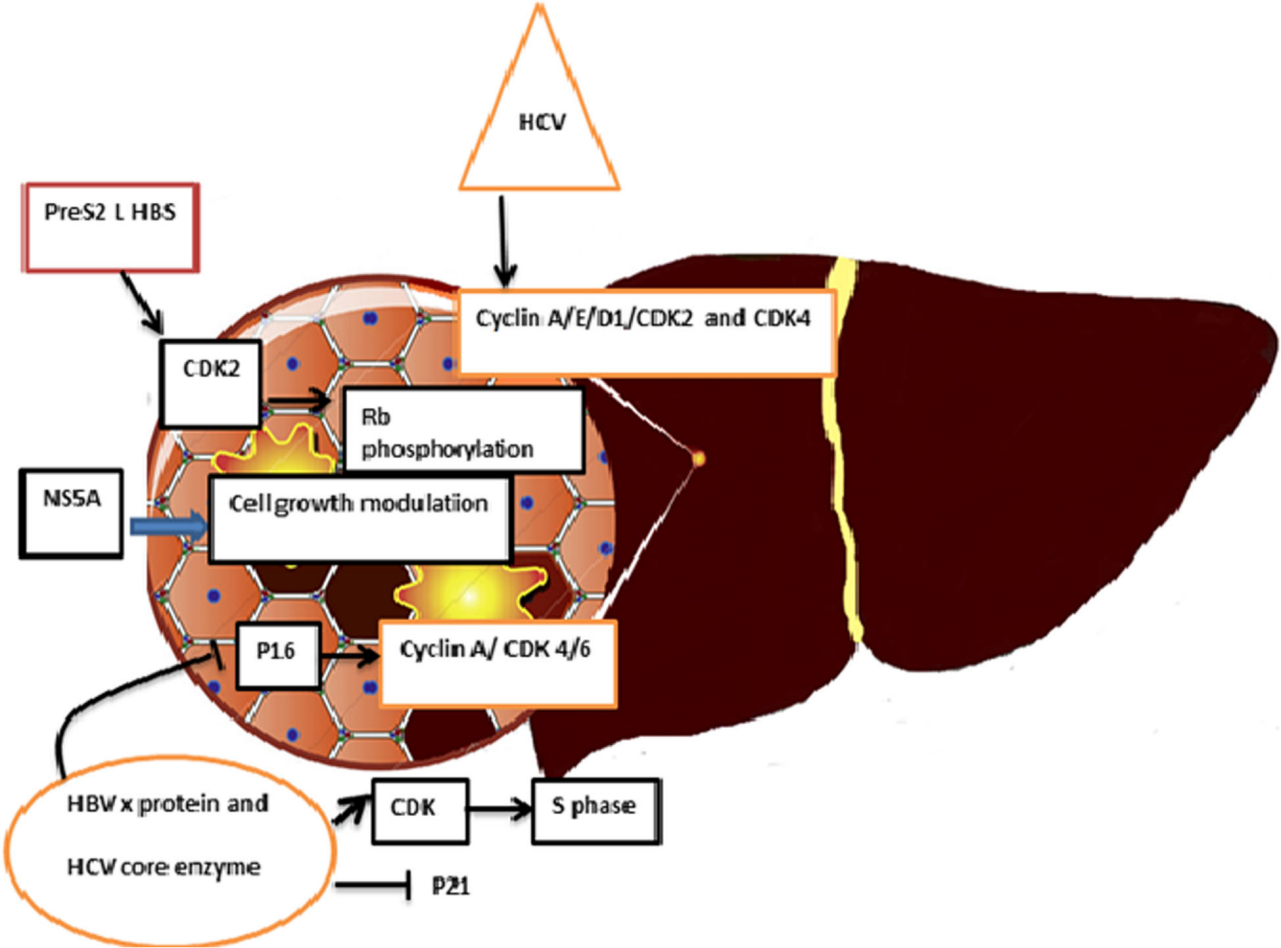
The regulation of cells CDK and CIKs expression by HBV and HCV

HBV also can express another onco-protein, called large surface antigen (LHBS). This protein is able to increase the phosphorylation of Rb, employs c-Jun activation Domain-Binding Protein 1 signaling factor to deregulate p27Kip1, and exhibits CDK2 activity [119]. The ability of this protein to activate CDK1 and CDK2 is similar to HBx protein [120]. It is worth to mention that HBx can induce p16 hypermethylation, CDK4/6 up-regulation, Rb phosphorylation, and E2F and DNMT1 activity [73] (Fig. 5) (Table 1).

## Human hepatitis C virus (HCV)

Hepatitis C virus (HCV), one of the major causes of hepatitis and liver cancer, possesses a positive-strand RNA that can regulate the expression level of CDK-activating kinase (CAK) [121, 122]. Although HCV could express wide range of proteins, its core protein possesses the most ability to interfere with the progression of the cell cycle. More than 10 HCV core proteins has been identified with the ability to modulate apoptosis pathways, as well as diverse biological activities [123]. Some studies suggested that this protein can inhibit the expression of CDK7. In addition, HCV could impair the cell cycle machinery through suppressing of CDK-activating kinase (CAK), CDK2 and CAK complex [74]. By cooperating with HBV, HCV core protein decreases the expression of p21 through influencing on the transforming growth Factor-L responsive element and Sp1 site of the p21 promoter [124]. The increase in the expression level of cyclinA, E, D1, CDK2 and CDK4, in the patients who are infected with HCV, could be exploit as a biomarker for the detection of hepatitis cancerous cells [75]. Another protein encoded by HCV which may have an impact on the regulation of CDK and CIK is NS5A. This protein increases the expression pf p21, as well [76]. In HCV infected patients, there is also an increase in the expression of CDK inhibitors, including p16 and p57 [77, 78] (Fig. 5) (Table 1).

## Reovirus

**Fig. 6:**
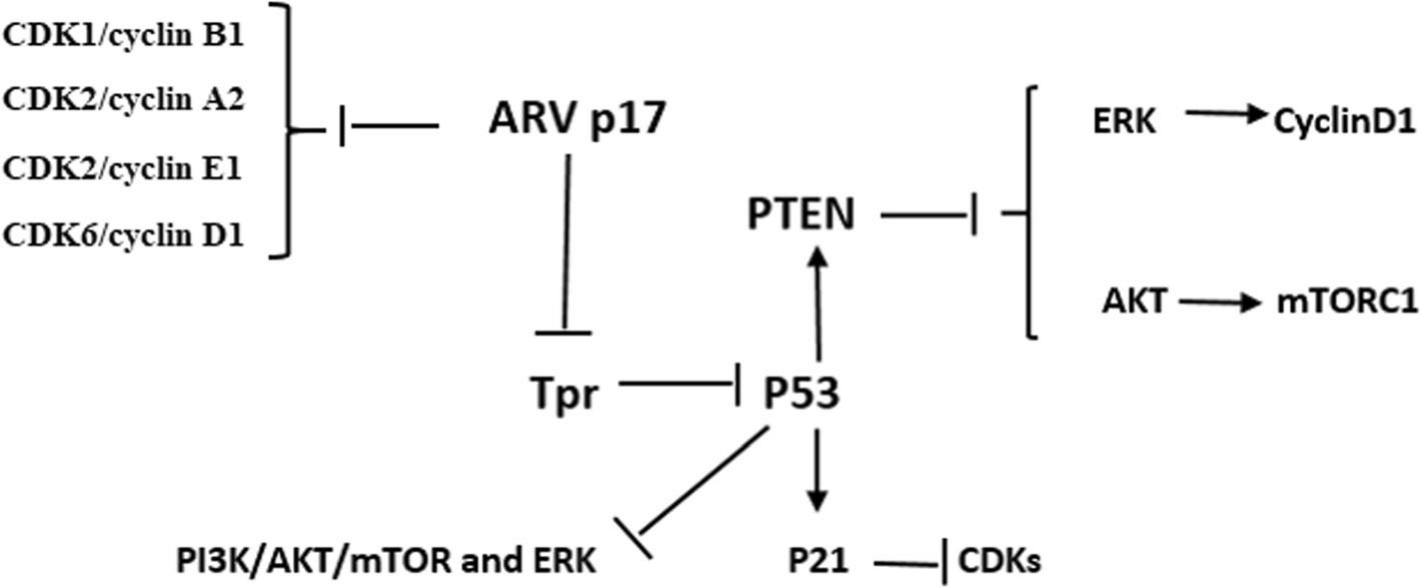
The effect of P17 reovirus on cancerous signaling pathway

Respiratory Enteric Orphan virus, commonly known as the reovirus, is an oncolytic virus. Both mammalian [125–127] and avian reoviruses [28, 79, 80] are under evaluation as cancer potential therapeutics. Current efforts focus on increasing the intrinsic capacity of mammalian reovirus to kill cancer cells, thwart cell division, optimizing the efficacy of reovirus combination therapies, and evaluating the effect of reovirus on immunotherapy [125–127]. However, a recent report suggested that avian reovirus p17 protein is a virus-encoded CDK inhibitor (v-CDKI). The ARV p17 protein suppresses CDK1/cyclin B1, CDK2/cyclin A2, CDK2/cyclin E1, and CDK6/cyclin D1 complexes by direct binding to CDKs, cyclins or complexes. In addition, the ARV p17 protein suppresses the CDK7/cyclin H complex by enhancement of p53 and cyclin H interaction [28, 80]. Furthermore, avian reovirus p17 protein has been demonstrated to function like v-CDKI, [28] which shuttles between the cytoplasm and the nucleus [128] to perform specific duties in mediating cancer cell cycle and growth [28]. Avian reovirus protein p17 is a nucleoporin Tpr Suppressor and can activate p53, p21 and PTEN and inactivates the PI3K/AKT/mTOR and ERK signaling pathways [79, 129]. In fact, the ARV p17 protein suppresses Tpr, leading to activation of p53, thereby activating p21 and PTEN as well as suppression of the PI3K/AKT/mTOR and ERK signaling pathways [129]. Also p17 may change some cell cycle pathway [28, 130, 131], such as, triggering autophagy by activating phosphatase [79, 132], altering cellular translation and so forth [133]. (Fig. 6).

## Conclusion

Dysregulation of the cell cycle through alteration in the expression CDK and CIKs has long been considered as a classic hallmark of cancer growth. Given to the importance of CDK and CIKs, we allocated this study to evaluate the effect of viral onco-proteins on the expression level of these proteins. Overviewing these mechanisms can highlight not only the management strategies but also the therapeutic approaches against these viral infections.

## Data Availability

Please contact author for data requests.

## Abbreviations

CDKs: Cyclin-dependent kinases
CKIs: Cyclin-dependent inhibitors
HPV: Human papillomaviruses
HERVs: Human endogenous retroviruses
EBV: Epstein-Barr virus
HCV: Hepatitis C virus
HBV: Human hepatitis B virus
KSHV: Herpes virus type 8
HBx: HBV X protein
LHBS: Large surface antigen
LMP1: Latent membrane protein 1
HTLV-1: Human T cell leukemia virus type 1
HTLV: Human T-lymphotropic viruses
